# Protective effect of voluntary medical male circumcision against sexually transmitted infections among adult men in Malawi: insights from observational data

**DOI:** 10.3389/fpubh.2026.1831372

**Published:** 2026-06-17

**Authors:** Tsirizani Mwalimu Kaombe

**Affiliations:** Department of Mathematical Sciences, School of Natural and Applied Sciences, University of Malawi, Zomba, Malawi

**Keywords:** HIV, mixed-effect logistic regression, sexually transmitted infections (STIs), survey data, voluntary medical male circumcision (VMMC)

## Abstract

**Background:**

Voluntary medical male circumcision (VMMC) has been shown to effectively reduce the risk of contracting Human Immunodeficiency Virus (HIV) and other sexually transmitted infections (STIs) in men. However, its implementation in sub-Saharan Africa faces several challenges, including low awareness and persistent cultural myths, which contribute to low participation rates. To improve the understanding of this intervention, further empirical evidence is needed regarding its protective effects.

**Methods:**

This article applied a mixed-effects logistic regression model to examine the adjusted impact of VMMC on self-reported STIs among men in Malawi, using data from the 2024 Malawi Demographic and Health Survey. The analyses were conducted using R software version 4.5.3.

**Results:**

The results revealed significant variation in HIV and STI outcomes across different clusters, indicating similarities inmen's sexual behaviors within communities. The protective effect of VMMC on STIs was selective; it was more effective against HIV infection than against other STIs. Medically circumcised men had a lower risk of HIV infection compared to uncircumcised men, but the effect size for other STIs was minimal. Factors such as being married or divorced, having multiple sexual partners, residing in urban areas, and belonging to the *Lomwe, Yao*, or *Sena* tribes were associated with an increased risk of HIV or STIs.

**Conclusion:**

The study recommends that health policymakers in sub-Saharan Africa focus VMMC campaigns on these high-risk subpopulations and involve men's spouses and local leaders to achieve better results from the intervention.

## 1 Introduction

Sexually transmitted infections (STIs) refer to all forms of irregular multiplication of harmful microorganisms in a host, chiefly initiated through unprotected sexual contact (1). These include human immunodeficiency virus (HIV), gonorrhea, hepatitis C virus (HCV), chlamydia, and syphillis (2, 3). STIs are typically spread through the exchange of contaminated fluids, such as semen, blood, vaginal fluid, rectal fluid, and breast milk, during unprotected sexual activity, blood transfusions, or from mother to child (4). The prevention of STIs can be approached through behavioral methods that promote safe sex practices, such as condom use, biomedical strategies that enhance the body's resistance to infection, and structural support mechanisms that facilitate prevention campaigns (4, 5). One effective biomedical strategy recommended for STI prevention is voluntary medical male circumcision (VMMC) (6).

Medical male circumcision is the surgical removal of the foreskin from the penis (7). Three randomized controlled trials conducted in Kenya, South Africa, and Uganda early 2000 by the National Institutes of Health (NIH) all provided evidence that VMMC reduces the risk of heterosexual HIV and other STIs acquisition in sexually active men by about two-thirds (7). The VMMC protection comes from the removal of the vulnerable region of entry for the HIV and related pathogens and promoting a less inflammatory residual penile microbiome (8, 9). There is also evidence of low risk of contracting chancroid and syphilis in circumcised men (7, 10). However, there is generally low uptake of voluntary medical male circumcision in sub-Saharan Africa, despite a high burden of STIs in the region (11). This is due to a lack of awareness about the health benefits of VMMC and cultural myths that associate circumcision with diminished sexual drive and performance (11, 12). The voluntary medical male circumcision (VMMC) differs from traditional male circumcision (TMC) in that TMC is typically performed as part of cultural or religious practices and is not conducted by medical professionals (13). The objectives of these two types of circumcision also differ. Traditional male circumcision is often seen as a rite of passage, marking the transition from childhood to youth and establishing social status within the community. In contrast, VMMC is a public health intervention aimed at reducing the risk of sexually transmitted infections (12).

Volunteering to get medically circumcised can improve the health of sexually active uncircumcised males and their partners (14). In addition to reducing the risk of sexually transmitted infections (STIs), male circumcision is thought to enhance sexual pleasure and improve penile hygiene (15). Further, male circumcision is known to reduce the risk of cervical cancer, bacterial vaginosis, Trichomonas vaginalis, and oncogenic human papillomavirus (HPV) genotypes, as well as other STIs in female partners (7, 16). The level of voluntariness in the VMMC programme in Sub-Saharan Africa is under debate. Some reports suggest that participants are often influenced by peer pressure, demands from female sexual partners, and persuasion from medical personnel (17, 18). Providing more scientific evidence about the health benefits of voluntary medical male circumcision could help clear the cultural myths about this intervention, which can potentially encourage sexually active males to adopt this STI prevention method, ultimately reducing the prevalence of STIs in sub-Saharan Africa (19). This research applies mixed-effects binary logistic regression to examine the protective effect of voluntary medical male circumcision against self-reported sexually transmitted infections (STIs) outcomes in adult men in Malawi, based on data from the 2024 Malawi Demographic and Health Survey. Since VMMC complements other STI prevention strategies (16, 20), this study adjusts for other factors, such as having multiple sexual partners, when evaluating the effect of VMMC on STI outcomes. The study also accounts for the clustering effects of STI outcomes in the estimation, that could emanate from peer pressure on risky sexual behavior and other unknown factors.

The findings from this research will contribute to bridging the knowledge gap among adult males in Malawi concerning STI prevention through VMMC (21). By offering the most current scientific evidence from a nationwide dataset, this local data will empower adult men in the region to make informed decisions about their participation in medical male circumcision programme in future. Furthermore, the results will assist health policymakers in crafting appropriate policies based on up-to-date information regarding VMMC.

This paper is organized as follows: Section 2 presents the data and statistical methods used, and Section 3 gives the results. This is followed by the discussion of findings in Section 4, and the conclusion in Section 5.

## 2 Methods

### 2.1 Data

The study used data from the male recode file of the 2024 Malawi Demographic and Health Survey (MDHS) to estimate the effect of VMMC on STI and HIV outcomes, while accounting for other factors. The survey was conducted between 12 May and 31 August 2024 by the National Statistical Office (NSO) of Malawi with the technical support from the ICF and DHS programme of the United States of America. The aim of the 2024 MDHS was to provide an up-to-date estimates regarding the basic demographic and health indicators in Malawi. The survey was a two-stage cluster-stratified survey, that randomly sampled 769 clusters in the first stage from list of all census enumeration areas (EAs) created for the 2018 Malawi housing and population census, and 23,070 households during the second stage (22). A subsample of these households were selected for men's interviews. A total of 8,913 men aged 15–54 years were studied.

Two dependent variables were used in this study. The first was whether the male respondent had any sexually transmitted infection (STI) in the last 12 months prior to the survey (no/yes). This included all types of diseases transmitted through sexual contact, such as abnormal penile discharge, or having genital sores or ulcers. The missing values were assigned the “no” option during data analysis, as it was a small proportion of the data and this did not affect the distribution of the STI variable (21). The second outcome variable was result of the most recent HIV test (negative or positive). The participants who declined to reveal their result or had missing responses were assigned the “negative” outcome category during data analysis, for similar reason as above. The regression analysis involved two separate models as opposed to joint/paired outcome models, since the reference times of observation for the two variables were different. A most recent HIV test result was not entirely referring to the previous 12 months, but even beyond. The primary independent variable in each case was the circumcision status of respondents, categorized as not circumcised, traditionally circumcised, or medically circumcised. Other explanatory variables included in the modeling, based on literature, were the number of sexual partners (excluding spouse) in the last 12 months, marital status, and religious affiliation (23, 24). Additionally, individual's current age, internet usage, knowledge of HIV (ever tested), ethnicity, education level, and place of residence were included in the model due to their previously reported associations with STI outcomes (25, 26).

The 2024 MDHS data (22) analyzed during this research are publicly available for users through the following link: https://www.dhsprogram.com/data/dataset/Malawi_Standard-DHS_2024.cfm?flag=1. Data cleaning was carried out using the STATA package version 12.0.

### 2.2 Mixed-effects logistic regression model and its estimation

Let *Y*_*ij*_ be a binary random variable capturing whether the *i*-th male respondent staying in cluster *j* self-reported to have had sexually transmitted infection in the 12 months preceding the survey or reported to have tested positive in most recent HIV test, with *y*_*ij*_ being its actual outcome. That is, *y*_*ij*_ = 1 for the respondent who expereinced any STI during that period or had tested HIV positive in recent test, and *y*_*ij*_ = 0 otherwise. Further, let θ_*ij*_ be the probability that the *ij*-th man experienced STI during the last 12 months of the survey or testing HIV positive during recent test, thus, π_*ij*_ = *P*(*Y*_*ij*_ = 1). In this case, *Y*_*ij*_ can be considered as a multivariate Bernoulli distributed random variable, where π_*ij*_ is its paramater, i.e., *Y*_*ij*_~*Bernoulli*(π_*ij*_) (27, 28). If π_*ij*_ was constant from one male respondent to the other for men sampled from the same cluster, then one can use mixed-effects logistic regression to estimate it from the independent variables *X*_*ij*_ described in Section 2.1 and some unknown cluster-based random covariates *Z*_*j*_ accounting for dependences of the outcomes within the same cluster (29, 30).

The mixed-effects logistic regression model is given by:


$$\begin{array}{l} Y_{ij}=\pi_{ij}\left(X_{ij},Z_{j}\right)+\epsilon_{ij}, \end{array}$$ (1)

where


$$\begin{array}{l} \pi_{ij}\left(X_{ij},Z_{j}\right)=\frac{1}{1+\exp\left(-\left(X_{ij}^{T}\beta +Z_{j}^{T}b_{j}\right)\right)}. \end{array}$$ (2)

The STI outcomes *Y*_*ij*_ in model Equations 1, 2 are assumed to be conditionally independent observations for each male respondent in each cluster. While the independent variables $X_{ijh}^{T}=\left(1,X_{ij1},X_{ij2},...,X_{ijp}\right)$ are considered systematically measured. The constants $\beta ={\left(\beta_{0},\beta_{1},\beta_{2},...,\beta_{p}\right)}^{T}$ are regression coefficients capturing the fixed effects of the variables *X*_*ij*_ on the outcome *Y*_*ij*_ in the model. The variables $Z_{j}^{T}=\left(Z_{1},Z_{2},...,Z_{q}\right)$ are cluster-level random covariates, with $b_{j}={\left(b_{1},b_{2},...,b_{q}\right)}^{T}$ being their respective effects. These random effects, *b*_*j*_'s were assumed to follow a multivariate normal distribution with mean **0** and covariance *D* (27, 31). The term ϵ_*ij*_ represented the model random error for observing the outcome in each respondent. The error term was assumed to have multivariate logistic distribution with mean of zero (32). The non-linear model in Equations 1, 2 can be linearised by taking logarithm of the ratio of the probabilities π_*ij*_(*X*_*ij*_, *Z*_*j*_) and 1−π_*ij*_(*X*_*ij*_, *Z*_*j*_) from Equation 2, as follows:


$$\begin{array}{l} \log\left(\frac{\pi_{ij}\left(X_{ij},Z_{j}\right)}{1-\pi_{ij}\left(X_{ij},Z_{j}\right)}\right)=X_{ij}^{T}\beta +Z_{j}^{T}b_{j}. \end{array}$$ (3)

The expression on the left hand side of Equation 3 is called the logarithm of odds (or logit) link function for a class of binary logistic regression models. It is precisely linking the assumed fixed probability of contracting STI or HIV in each individual, i.e., (*P*(*Y*_*ij*_ = 1)), to a linear combination of fixed and random explanatory variables without any error of measurement (32). All interpretations of the regression coefficients for the model are made in reference to the log odds and their variation between clusters (29).

#### 2.2.1 The maximum likelihood estimation function for the mixed-effects logistic regression model

The fixed and random effects model parameters in Equations 1–3 were estimated through the penalized quasi-likelihood method (27, 33). The method considers the distribution function of the random effects as a penalty term in the log-likelihood function, when estimating fixed effects. Thereafter, the results of the fixed effects estimates are used to complete the estimation of random effects in the likelihood function (33). If *Z*_*j*_ is unity across clusters, and $b_{j}\sim N\left(0,\sigma^{2}\right)$, and considering the assumed Bernoulli distribution for the dependent variable, the penalized quasi-likelihood function for the model is given by:


$$\begin{array}{l} L\left(\beta ,\sigma^{2}\right)=\overset{M}{\underset{j=1}{\prod }}\overset{n_{j}}{\underset{i=1}{\prod }}\left[{\left(\pi_{ij}\left(X_{ij},Z_{j}\right)\right)}^{y_{ij}}{\left(1-\pi_{ij}\left(X_{ij},Z_{j}\right)\right)}^{\left(1-y_{ij}\right)}\right]\\ \;\times {\left(\frac{1}{\sigma \sqrt{2\pi }}\right)}^{M}\exp\left(-\frac{M}{2\sigma^{2}}\overset{M}{\underset{j=1}{\sum }}b_{j}^{2}\right). \end{array}$$ (4)

Considering the expression given in Equation 2, the likelihood function in Equation 4 is simplified to:


$$\begin{array}{c} L\left(\beta ,\sigma^{2}\right)=\prod_{j=1}^{M}\prod_{i=1}^{n_{j}}\\ \left[{\left(\frac{1}{1+\exp\left(-\left(X_{ij}^{T}\beta +Z_{j}^{T}b_{j}\right)\right)}\right)}^{y_{ij}}{\left(1+\exp\left(X_{ij}^{T}\beta +Z_{j}^{T}b_{j}\right)\right)}^{-\left(1-y_{ij}\right)}\right]\\ \times {\left(\frac{1}{\sigma \sqrt{2\pi }}\right)}^{M}\exp\left(-\frac{M}{2\sigma^{2}}\sum_{j=1}^{M}b_{j}^{2}\right). \end{array}$$ (5)

To estimate of the parameters β and σ^2^, the log-likelihood function was used. This was obtained by taking the logarithm of the likelihood function in Equation 5 and simplifying it to get the following function:


$$\begin{array}{l} l\left(\beta ,\sigma^{2}\right)=\overset{M}{\underset{j=1}{\sum }}\overset{n_{j}}{\underset{i=1}{\sum }}(Y_{ij}\left(X_{ij}^{T}\beta +Z_{j}^{T}b_{j}\right)-\log\left(1+\exp\left(X_{ij}^{T}\beta +Z_{j}^{T}b_{j}\right)\right)\\ \;+Mlog\left(\frac{1}{\sigma \sqrt{2\pi }}\right)-\frac{M}{2\sigma^{2}}\overset{M}{\underset{j=1}{\sum }}b_{j}^{2}. \end{array}$$ (6)

The estimates were found by equating to zero the first partial derivatives of the log-likelihood function in Equation 6 with respect to β, σ^2^ and solve for the parameters. The process was done with the help of numerical methods as the zero equations were not in closed form (27). The maximum likelihood (ML) estimates $\hat{\beta }$ represented a change in the logarithm of the odds of contracting STI or HIV corresponding to a unit change in the covariate *X*. While ${\hat{\sigma }}^{2}$ indicated amount of variation in the log-odds for model intercepts from different clusters. A zero variance showed no variation of the intercepts, while the value larger than zero indicated significant variation in intercept estimates. The R package *lme4* (34) in the R software version 4.5.3 environment (48) was used for all model computations. The R code involved during the analysis is given as an Appendix 1. Prior to model fitting, frequencies of HIV and STI against each independent variable, as well as Chi-square test results were used to summarize the data.

### 2.3 Model selection

Owing to the fact that the research involved a variety of explanatory variables, as indicated in Section 2.1, it was important to make conclusions with few that were useful. The variables with large p-values and standard errors were candidates for removal in the final model. The selection of variables was done using the Akaike information criterion (35, 36) given by:


$$\begin{array}{l} AIC=2p-2l\left(\hat{\beta }\right), \end{array}$$ (7)

where *p* in Equation (7) is the number of fixed regression constants, and $l\left(\hat{\beta }\right)$ is the estimate of the log-likelihood function. Given various subsets of independent variables included in the model, the model with smallest AIC was considered as the best to proceed with in the final conclusions (35).

## 3 Results

### 3.1 Data summary

The data summary presented in Table 1 indicates that sexually transmitted infections (STIs) and HIV were reported in a minority of respondents, specifically 5% and 3%, respectively. Both conditions were most common among men who were identified as having no religion, those who were separated, divorced, or widowed, and those who were either traditionally circumcised or uncircumcised. Additionally, higher rates of STIs and HIV were observed in men who did not use the internet and those who had ever been tested for HIV. STIs were particularly prevalent among men from the Chewa and Mang'anja tribes, those with only a primary education, and those who had at least two sexual partners outside of their spouses. In contrast, HIV was most commonly found in men from the *Lomwe, Yao*, and *Sena* ethnic groups, those with no education, and those who had no more than one sexual partner.

**Table 1:** Distribution of self-reported STI and HIV Test results by the socio-demographic characteristics of the male respondents, 2024 MDHS.

	Had STI in last 12 months			Recent HIV test result	
Characteristic	*n* (%)	Yes (%)	χ^2^ *p*-value	Positive (%)	χ^2^ *p*-value
Overall sample	8,913 (100)	470 (05.3)		263 (03.0)	
Ethnicity			0.0290		< 0.0001
Tumbuka/Tonga/other	2999 (33.7)	138 (04.6)		67 (02.2)	
Lomwe/Yao/Sena	2776 (31.2)	141 (05.1)		129 (04.7)	
Chewa/Mang'anja	3138 (35.2)	191 (06.1)		67 (02.1)	
Religion			< 0.0001		0.7090
No religion/other	329 (03.7)	32 (09.7)		12 (03.7)	
Muslim	870 (09.8)	54 (06.2)		27 (03.1)	
Christian	7714 (86.6)	384 (05.0)		224 (02.9)	
Marital status			< 0.0001		< 0.0001
Never in union	3809 (42.7)	107 (02.8)		23 (00.6)	
Married/cohabited	4730 (53.1)	321 (06.8)		216 (04.6)	
Separated/other	374 (04.2)	42 (11.2)		24 (06.4)	
Education level			0.0400		0.0020
No education	413 (04.6)	19 (04.6)		24 (05.8)	
Primary	4916 (55.2)	277 (05.6)		137 (02.8)	
Secondary	3072 (34.5)	160 (05.2)		93 (03.0)	
Higher	512 (05.7)	14 (02.7)		9 (01.8)	
Circumcision Status			0.0100		< 0.0001
Not circumcised	5645 (63.3)	287 (05.1)		172 (03.0)	
Traditionally circumcised	1238 (13.9)	87 (07.0)		56 (04.5)	
Medically circumcised	2030 (22.8)	96 (04.7)		35 (01.7)	
Sex partners in last 12 months†			< 0.0001		0.3450
0	5963 (66.9)	219 (03.7)		181 (03.0)	
1	2205 (24.7)	171 (07.8)		68 (03.1)	
2	507 (05.7)	58 (11.4)		10 (02.0)	
3	238 (02.7)	22 (09.2)		4 (01.7)	
Internet usage			0.7130		0.0190
Never used	5827 (65.4)	314 (05.4)		193 (03.3)	
Used last 12 months	2629 (29.5)	135 (05.1)		58 (02.2)	
Used before last 12 months	457 (05.1)	21 (04.6)		12 (02.6)	
Ever been tested for HIV			< 0.0001		< 0.0001
No	2586 (29.0)	56 (02.2)		0 (00.0)	
Yes	6327 (71.0)	414 (06.5)		263 (04.2)	
Place of residence			0.9060		0.0440
Urban	2049 (23.0)	107 (05.2)		74 (03.6)	
Rural	6864 (77)	363 (05.3)		189 (02.8)	
Mean age (SD)	28.7 (10.9)				

SD, standard deviation; χ^2^ *p*-valule, Chi-square test. † sex partners excluded spouse.

The Chi-square test results indicated that, at a 5% significance level, both sexually transmitted infections (STI) and HIV were significantly associated with ethnicity, marital status, education level, circumcision status, and whether individuals had been tested for HIV. In contrast, religion, and the number of sexual partners in the past 12 months were only associated with STIs and not with HIV. Similarly, place of residence and internet usage were linked only to HIV and not to STIs. The average age of the respondents was 29 years, with a standard deviation of 11 years.

### 3.2 Effects of male circumcision and other variables on STIs and HIV test results based on the mixed-effects logistic regression model estimates

The results presented in Table 2 show the maximum likelihood (ML) estimates from separate mixed-effects logistic regression models analyzing STI and HIV data from the 2024 MDHS. In both models, the variance of the cluster random effects for the model intercepts was significantly different from zero. This indicates that the log-odds of contracting STIs and HIV varied considerably between clusters, making it crucial to account for this variation in the analysis. Additionally, the AIC estimates revealed that models incorporating other variables while assessing the effect of male circumcision on STIs and HIV provided a better fit to the data compared to models where circumcision status was the only variable considered.

**Table 2:** Adjusted effects of circumcision status and other male characteristics on STI and HIV outcomes upon fitting mixed-effects logistic regression model to 2024MDHS data.

	STI in last 12 months			Recent HIV test result		
	Model 1	Model 2	Model 3	Model 1	Model 2	Model 3
Variable	OR (SE; *p*-val)	OR (SE; *p*-val)	OR (SE; *p*-val)	OR (SE; *p*-val)	OR (SE; *p*-val)	OR (SE; *p*-val)
Constant	0.01 (0.43; < 0.001)	0.01 (0.26; < 0.001)	0.04 (0.09, < 0.001)	0.00 (75.5; 0.745)	0.00 (0.36; < 0.001)	0.03 (0.11; < 0.001)
Ethnicity						
Tumbuka/Tonga/ot^*^						
Lomwe/Yao/Sena	0.92 (0.16; 0.572)	0.99 (0.15; 0.954)		2.21 (0.17; < 0.001)	2.23 (0.17; < 0.001)	
Chewa/Mang'anja	1.20 (0.14; 0.189)	1.28 (0.14; 0.066)		0.93 (0.19; 0.705)	0.96 (0.18; 0.840)	
Religion						
**No religion/other** ^*^						
Muslim	0.69 (0.30; 0.216)			0.58 (0.41; 0.187)		
Christian	0.58 (0.22; 0.013)			0.83 (0.33; 0.558)		
Marital status						
**Never in union** ^*^						
Married/cohabited	3.74 (0.17; < 0.001)	4.71 (0.16; < 0.001)		0.99 (0.28; 0.959)	1.74 (0.28; 0.045)	
Separated/other	3.43 (0.22; < 0.001)	4.09 (0.22; < 0.001)		1.31 (0.34; 0.427)	2.11 (0.34; 0.027)	
Education level						
**No education** ^*^						
Primary	1.61 (0.26; 0.070)			0.88 (0.25; 0.608)		
Secondary	1.44 (0.28; 0.192)			0.96 (0.27; 0.873)		
Higher	0.69 (0.40; 0.350)			0.43 (0.45; 0.057)		
Circumcision Status						
**Not circumcised** ^*^						
Trad. circumcised	1.17 (0.18; 0.367)	1.29 (0.15; 0.100)	1.48 (0.14; 0.005)	1.02 (0.20; 0.913)	0.93 (0.18; 0.666)	1.47 (0.16; 0.017)
Med. circumcised	0.90 (0.14; 0.474)	0.92 (0.14; 0.555)	0.90 (0.13; 0.400)	0.75 (0.21; 0.157)	0.76 (0.20; 0.172)	0.54 (0.19; 0.001)
**Sex partners** †						
**0** ^*^						
1	3.28 (0.12; < 0.001)	3.43 (0.12; < 0.001)		1.77 (0.17; 0.001)	1.87 (0.16; 0.0001)	
2	5.19 (0.18; < 0.001)	5.45 (0.18; < 0.001)		1.29 (0.35; 0.474)	1.42 (0.35; 0.315)	
3	5.81 (0.28; < 0.001)	6.32 (0.27; < 0.001)		2.09 (0.55; 0.184)	2.42 (0.55; 0.111)	
Internet usage						
**Never used** ^*^						
Used last 12 mths	0.94 (0.13; 0.646)			0.70 (0.19; 0.060)		
Used b4 last 12 ms	0.69 (0.25; 0.135)			0.93 (0.32; 0.824)		
Ever had HIV test						
**No** ^*^						
Yes	2.06 (0.17; < 0.001)			8.8x10^7^ (76; 0.809)		
Place of residence						
**Urban** ^*^						
Rural	0.78 (0.16; 0.105)	0.86 (0.15; 0.294)		0.62 (0.16; 0.003)	0.72 (0.15; 0.026)	
Current age	1.00 (0.01; 0.400)	1.00 (0.01; 0.531)		1.10 (0.01; < 0.001)		
Var (RE-Interc)	0.7282	0.7283	0.7116	0.0779	0.0594	0.2521
AIC	3370.9	3394.3	3630.7	1935.7	2008.6	2355.1

SE, standard error; RE, random effects; (*), reference category; OR, odds ratio; †sex partners in last 12 months, excluding spouse.

Both the full model (Model 1) and the reduced models (Models 2 and 3) indicated that medical circumcision is associated with a decreased likelihood of contracting either an STI or HIV. The effect size for HIV was significantly greater than that for STIs. Specifically, the odds of contracting HIV were reduced by 25% to 46% in men who were medically circumcised compared to those who were not. This finding was highly statistically significant in the unadjusted model (Model 3), which reported a 46% reduction in odds. In contrast, the models showed that medically circumcised men had about a 10% lower likelihood of contracting any STI compared to uncircumcised men, although this difference was not statistically significant. Conversely, men who underwent traditional circumcision had higher odds of contracting both STIs and HIV compared to their uncircumcised counterparts. While the differences were not statistically significant overall, the unadjusted HIV model (Model 3) did show a 47% increase in odds for this group.

In general, disregarding any covariates in the model, the odds of contracting an STI or HIV were over 95% lower in the male population. The likelihood of being infected with HIV was twice as high in *Lomwe, Yao*, and *Sena* males compared to *Tumbuka, Tonga*, and related tribes, after controlling for other variables in the model. There was no difference in the risk of HIV between *Chewa* or *Mang'anja* and *Tumbuka* or *Tonga* men (Models 1 and 2). When it comes to STIs, the risk was over 28% higher in Chewa and Mang'anja men compared to Tumbuka and Tonga men (Model 2). No difference in STI risk was found between *Lomwe, Yao*, or *Sena* and *Tumbuka* or *Tonga* men. Additionally, Christian men had a 42% lower likelihood of contracting STIs compared to men with no religion (Model 1). No difference was observed between Muslims and non-religious men, and there was no association between HIV and religion.

Married, cohabiting, separated, divorced, or widowed men had approximately four times higher odds of contracting sexually transmitted infections (STIs) (Models 1 and 2) and about twice the odds of acquiring HIV (Model 2) compared to men who had never been in a union. Men with higher education levels had a 57% reduced likelihood of contracting HIV (Model 1). There was no significant difference in the risk of STIs among men with varying education levels; those with primary and secondary education had similar risks to those with no education. Additionally, the risk of HIV was nearly twice as high in men who have had at least one sexual partner outside of their marriage compared to those who have not. Likewise, the odds of contracting an STI were five times greater in men with at least one sexual partner than in those with none.

The risk of both HIV and sexually transmitted infections (STIs) was found to be low among men who used the internet compared to those who never used it, although the difference was not statistically significant. Men who had ever tested for HIV had twice the odds of contracting an STI compared to those who had never been tested. However, testing for HIV showed no association with contracting HIV itself. Additionally, men living in rural areas had a 30% to 40% lower likelihood of contracting HIV compared to those residing in urban settings. The risk of contracting STIs was also low among men from rural areas, though this difference was not significant at the 5% level. Increased age of an individual was associated with a 10% higher odds of contracting HIV.

## 4 Discussion

This study analyzed the adjusted effects of voluntary medical male circumcision (VMMC) on sexually transmitted infections (STIs) and HIV outcomes using survey data to provide evidence supporting regional VMMC programmes. The analysis utilized a large nationwide sample of 8,913 men from the 2024 Malawi Demographic and Health Survey. The results indicated that STI and HIV outcomes varied significantly between different clusters. This confirmed previous observations that risky sexual behavior among men may be influenced by peer pressure within their neighborhoods, among other factors (17, 18). Therefore, it is crucial to account for such clustering of behaviors when analyzing health outcomes to avoid biased estimates (30). Moreover, the study found that voluntary medical male circumcision was more effective in reducing the risk of HIV infection compared to other STIs. The risk of HIV infection in medically circumcised males was notably lower than that associated with other STIs. These findings suggest that while VMMC is effective against HIV, its effectiveness may not extend to all STIs, particularly those that do not involve the entry of pathogens into the bloodstream through the genital area (7, 9, 37). Including various types of STIs in this study, such as genital sores and ulcers, may have obscured the protective effect of VMMC against certain STIs (6).

The findings revealed that voluntary medical male circumcision (VMMC) offers a superior protective effect compared to traditional circumcision. The risk of sexually transmitted infections (STIs) and HIV in traditionally circumcised men was either higher than or equal to that of uncircumcised males. In contrast, VMMC significantly reduces this risk. This difference may be due to the broader area of protected penile skin that VMMC covers, which is often not fully addressed in traditional circumcision practices (9). Additionally, several factors influenced the likelihood of contracting HIV or STIs, including ethnicity, marital status, the number of sexual partners, and place of residence. The risk of HIV was notably higher among men from the *Lomwe, Yao*, and *Sena* ethnic groups compared to those from the Tumbuka and Tonga tribes. This discrepancy may reflect risky cultural sexual behaviors prevalent in the former groups, such as *fisi* (unidentified sexual partner), *kulowakufa* (sexual inheritance), and *kusasa fumbi* (sexual cleansing), which have been reported to be common in these communities (38).

Married, cohabiting, divorced, widowed, and separated men had a higher risk of sexually transmitted infections (STIs) and HIV compared to unmarried men. Previous studies have attributed this increased risk to reckless sexual behaviors, such as extramarital relationships, particularly among married or divorced individuals (39, 40). Additionally, having a higher number of sexual partners is associated with an increased risk of both STIs and HIV, a finding that has been confirmed by several earlier studies (23, 24). The presence of multiple sexual partners creates a transmission network for HIV and STIs (41). Interestingly, this study found that men from rural areas had a lower risk of contracting both HIV and STIs compared to those living in urban settings. This finding was unexpected, as it was assumed that rural individuals would have lower exposure to media and prevention messages related to HIV and STIs, coupled with the effects of poverty (42). However, the increased risk in urban areas may be linked to a higher prevalence of commercial female sex work and other risky sexual practices, which expose men to a greater likelihood of contracting STIs and HIV (43).

One limitation encountered in this study was that the assessed outcomes for STIs and HIV did not correspond to the same period of observation. The STI responses covered a period of 12 months preceding the survey, while the HIV test results referred to any recent tests that participants had undergone. Although most respondents had HIV tests within the last 2 years prior to the survey, some reported results from much earlier. This discrepancy limited the ability to use paired or bivariate logistic regression models, which could have accounted for the relationship between the two dependent variables in the analysis (44). Additionally, no causal analyzes were performed due to the very small number of cases of HIV and STIs (at most 5% of the sample). Conducting causal inference matching analyzes could have further reduced the sample size for HIV and STIs, potentially biasing the model estimates (45–47). Nonetheless, a causal relationship between voluntary medical male circumcision (VMMC) and HIV has already been established through clinical trials conducted in Kenya, South Africa, and Uganda (7). This research aimed to generate additional evidence through alternative statistical methods to support the implementation of VMMC programmes in sub-Saharan Africa, where the burden of HIV is high and myths surrounding VMMC are widespread (11).

## 5 Conclusion

This research examined the effect of voluntary medical male circumcision on self-reported STI and HIV outcomes, while accounting for data clustering and other influencing factors. A large sample of 8,913 men aged between 15 and 54 years, based on the 2024 Malawi Demographic and Health Survey, was used. The data revealed significant between-cluster variation in HIV and STI outcomes, indicating that peer pressure and neighborly relationships may influence men's sexual behaviors. This variation must be considered when analyzing such data. The findings showed a notable protective effect of voluntary medical male circumcision against HIV infection, while no similar effectiveness was observed for other STIs. Medically circumcised men exhibited a significantly lower risk of HIV infection compared to their uncircumcised counterparts; however, the effectiveness for preventing other STIs was less pronounced.

The study has found that voluntary medical male circumcision (VMMC) is more effective than traditional circumcision in reducing the risk of sexually transmitted infections (STIs) and HIV. There was no significant difference in the risk of contracting STIs or HIV between men who were traditionally circumcised and those who were uncircumcised; in fact, the risk was even higher among traditionally circumcised men. The findings indicate that certain groups are at a higher risk of HIV. Specifically, men from the Lomwe, Yao, and Sena ethnic groups, as well as those who were married, cohabiting, divorced, widowed, or separated, had an increased risk, especially if they had had at least one sexual partner outside of their primary relationship. Conversely, the risk was lower among men living in rural areas. The study recommends targeting VMMC initiatives toward high-risk subpopulations, including men in urban areas, those who are married or divorced/widowed, and individuals from the Yao, Lomwe, and Sena ethnic groups. Effective VMMC campaigns should involve awareness messages that engage men's spouses and local community leaders to encourage men to voluntarily undergo this surgical procedure. Future research could utilize longitudinal statistical methods to analyze panel survey data to establish the consistency of these findings. Additionally, combining national survey data from both men and women could facilitate the assessment of VMMC's effectiveness in reducing STIs that may affect their female partners, ultimately promoting the VMMC program.

## Data Availability

The datasets presented in this study can be found in online repositories. The names of the repository/repositories and accession number(s) can be found below: https://www.dhsprogram.com/data/dataset/Malawi_Standard-DHS_2024.cfm?flag=1.
