# Supplementary material for: Protective effect of voluntary medical male circumcision against sexually transmitted infections among adult men in Malawi: insights from observational data
Source: Front Public Health. 2026 Jun 17;14:1831372. doi: 10.3389/fpubh.2026.1831372 (PMC13318940; doi:10.3389/fpubh.2026.1831372)
Supplement: Supplementary file 1 [file Data_Sheet_1.pdf]

Appendix 1: R code used to fit mixed-effects logistic regression model for the VMMC article

```
rm(list=ls())

library(foreign)

library(lme4)

library(compiler)

library(parallel)

library(boot)

library(lattice)

library(car)

library(ggplot2)

library(reshape2)

library(nnet)

library(ggrepel)

library(dplyr)

library(data.table)

library(readstata13)


> dt24 = read.dta("C:/Users/User/Desktop/DHS and MICS Datasets/MWMR81FL.dta",convert.factors=F)


> model1 <- glmer(STI ~ as.factor(Residence) + as.factor(Education) +as.factor(Religion) +
as.factor(Ethnicity) + as.factor(Marital) +as.factor(Internet) + as.factor(CircumStatus) +
as.factor(mv766a) + as.factor(mv781) + mv012  +   (1 | mv001), data = dt24, family = binomial, control =
glmerControl(optimizer = "bobyqa"), nAGQ = 10)


> summary(model1)


> model1b <- glmer(HIV ~ as.factor(Residence) + as.factor(Education) +as.factor(Religion) +
as.factor(Ethnicity) + as.factor(Marital) +as.factor(Internet) + as.factor(CircumStatus) +
as.factor(mv766a) + as.factor(mv781) + mv012  +   (1 | mv001), data = dt24, family = binomial, control =
glmerControl(optimizer = "bobyqa"), nAGQ = 10)
```

```
> summary(model1b)

> model2 <- glmer(STI ~ as.factor(Residence) + as.factor(Ethnicity) + as.factor(Marital) +
as.factor(CircumStatus) + as.factor(mv766a) + mv012  +   (1 | mv001), data = dt24, family = binomial,
control = glmerControl(optimizer = "bobyqa"), nAGQ = 10)

> summary(model2)

> model2b <- glmer(HIV ~ as.factor(Residence) + as.factor(Ethnicity) + as.factor(Marital) +
as.factor(CircumStatus) + as.factor(mv766a) + mv012  +   (1 | mv001), data = dt24, family = binomial,
control = glmerControl(optimizer = "bobyqa"), nAGQ = 10)

> summary(model2b)

> model3 <- glmer(STI ~ as.factor(CircumStatus)  +   (1 | mv001), data = dt24, family = binomial, control =
glmerControl(optimizer = "bobyqa"), nAGQ = 10)

> summary(model3)

> model3b <- glmer(HIV ~ as.factor(CircumStatus)  +   (1 | mv001), data = dt24, family = binomial,
control = glmerControl(optimizer = "bobyqa"), nAGQ = 10)

> summary(model3b)
```
